# Effectiveness of green tea mouthwash in postoperative pain control following surgical removal of impacted third molars: double blind randomized clinical trial

**DOI:** 10.1186/2008-2231-21-59

**Published:** 2013-07-18

**Authors:** Majid Eshghpour, Hamed Mortazavi, Naser Mohammadzadeh Rezaei, AmirHossein Nejat

**Affiliations:** 1 Dental Research Center, Department of Oral and Maxillofacial, Mashhad University of Medical Sciences, Mashhad, Iran; 2 Department of Oral Medicine, Dental School, Shahid Beheshti University of Medical Sciences, Tehran, Iran; 3 Mashhad University of Medical Sciences, Mashhad, Iran; 4 Member of Student Research Committee, School of Dentistry, Mashhad University of Medical Sciences, Mashhad, Iran

**Keywords:** Camellia sinensis, Impacted third molar, Mouthwash, Oral surgery, Postoperative pain

## Abstract

**Background:**

Pain following surgical removal of impacted molars has remained an important concern among practitioners. Various protocols have been proposed to reduce postoperative pain. However, each one has special side effects and limitations. As green tea possesses anti-inflammatory and antibacterial properties, the aim of the current study was to evaluate the effectiveness of green tea mouthwash in controlling postoperative pain.

**Materials and methods:**

In a study with split-mouth and double blind design, 44 patients in need of bilateral removal of impacted third molars underwent randomized surgical extraction; following one surgery patients rinsed with a green tea mouthwash from the first to seventh postoperative day and after other extraction rinsed with placebo mouthwash in the same duration. Both patients and surgeon were blinded to the type of mouthwash. The predictor variable was type of mouthwash and primary outcome variable was postoperative pain measured by visual analogue scale (VAS) during first week after surgery. In addition, number of analgesics patients used after surgery recorded. To measure the effect of green tea mouthwash, repeated measures test with confidence interval of 95% was performed.

**Results:**

Total of 43 patients with mean age of 24 years underwent total of 86 surgeries. VAS value had no statistically difference prior rinsing among groups (P-value > 0.05). However, the mean value of VAS following rinsing with green tea was statistically lower than placebo in postoperative days of 3–7 (P-value < 0.05). In addition, while rinsing with green tea, patients took significantly lower number of analgesics after surgery (P-value < 0.05). No side effects reported.

**Conclusion:**

Green tea mouthwash could be an appropriate and safe choice to control postoperative pain after third molar surgery.

## Introduction

The main aim of dental practices is not only to provide appropriate treatment and restore function, but also is to remove pain and bring relief the to their patients [1]. On the other hand, changes in lifestyle have resulted in smaller human jaw and lack of enough space for third molar eruption [2]. Hence, incidence of third molar impaction has increased and postoperative pain following surgical removal of third molar teeth has remained an important concern in need of control for many practitioners [3,4]. In addition to the inconvenience, the perception of patient toward dental practice would change after pain experience [5,6].

Various medications have been used to control postoperative pain following surgical removal of impacted molar teeth [1,7-13]. Analgesics including paracetamole and diclofenac sodium in addition to non-steroidal anti-inflammatory drugs (NSAIDs) have been widely used to control postoperative pain. However, gastrointestinal and renal complications are of possible side effects reported for NSAIDs [14].

Green tea (Camellia Sinensis) has been a popular drink in eastern countries for many years. Green tea is very rich in polyphenols including catechins which possess antioxidant, antidiabetic, antimutagenic, antiviral, antibacterial, and anti-inflammatory properties [15]. It has reported that green tea is effective on periodontal diseases and is also beneficial against cariogenic activities [16-19]. However, there exists no study to investigate benefits of green tea extract in controlling postoperative complications in oral surgeries.

The purpose of this study was to address the following question: is green tea mouthwash effective in relieving the pain following surgical removal of impacted mandibular third molars? The investigators hypothesized that the pain following rinsing with green tea after surgical removal of mandibular impacted third molar would equal to the pain following rinsing with placebo mouthwash.

## Materials and method

This study was performed at Mashhad Oral and Maxillofacial Surgery Clinic. The Ethical Committee of Mashhad University of Medical Sciences approved the study protocol and all the patients provided a signed detailed informed consent.

### Study design

To investigate the research hypothesis, the investigators performed a split-mouth, randomized, double-blind study based on the consent statement and Declaration of Helsinki.

### Study sample

The study population consisted of 44 patients in need of bilateral impacted third molar surgery between April 2012 and September 2012.

The inclusion criteria were: be 18–30 years old; have bilateral mandibular impacted third molars; have moderate difficulty level of impacted teeth on both sides of mandible based on the sum score of values regarding the spatial direction of the teeth, depth of impaction, and relationship with the ramus on preoperative panoramic radiography (Table 1) [20].

**Table 1:** Measurement of difficulty level of impacted teeth according to panoramic radiograph

**Spatial**	**Depth**	**Ramus**	**Difficulty score**
**direction**	**(value)**	**relationship**	**(sum of values)**
**(value)**		**(value)**	
Mesioangular (1)	Level A (1)	Class I (1)	Minimally difficult (3–4)
Horizontal (2)	Level B (2)	Class II (2)	Moderate (5–7)
Vertical (3)	Level C (3)	Class III (3)	Very difficult (8–10)
Distoangular (4)			

Patients were excluded from the study if: were smoking, were lactating or pregnant, were using analgesic drugs, had received antibiotic during past 2 weeks, had systemic disorders, or had any lesions on panoramic radiography.

### Study variables

The predictor variable was type of mouthrinse used in the study and control groups (green tea or placebo mouthwash). The outcome variable was self-reported pain (based on VAS). Other study variables were demographic variables (including age and sex), surgical variables (including operation time and extraction difficulty score), and post surgical variables (including number of analgesics used during first postoperative week). Operation time defined as time between the first incision till flap closure.

### Mouthwash preparation

Green tea extract prepared in the pharmacology laboratory of Mashhad University of Medical Sciences with the following protocol: Camellia Sinesis leaves were dried in 40°C for 45 minutes and powdered with electrical mortar; 100 grams of powder mixed with 500 ml of water; After 48 hours this mixture was filtered and the sediment was removed; The remnant solution was stored in room temperature; After 4 days the powder of green tea extract was obtained.

To obtain the green tea mouthwash, 5 g of extracted solved in 100 ml distilled water to produce 5% mouthwash. The rinse poured into 250 ml dark bottles. The placebo mouthwash consisted 250 ml distilled water. In addition, the mint flavor added to both study and placebo rinses to make the rinse type unidentifiable for patients.

### Surgical procedure

All the surgeries performed by an experienced surgeon using the same protocol: povidone iodine solution was applied around the mouth; 2% lidocaine + 1:80,000 epinephrine carpules were used to block the inferior alveolar/long buccal nerves; a mucoperiosteal envelop flap was created using a standard incision; if needed, bone removal, tooth sectioning, and bone recontouring were performed with a low-speed handpiece under sufficient sterile solution irrigation; following tooth removal the socket was irrigated with 60 ml of saline; the flap was sutured using 3–0 silk sutures. Patients were instructed to take two pills of Acetaminophen (325 mg) one prior the surgery and another 4 h after surgery. From the next day, patients instructed to take analgesics (Acetaminophen) after VAS assessment and also to record time and number of analgesics they had taken.

### Patient allocation

Following each surgery, patients received a bottle containing 250 ml mouthwash. Each patient received both green tea extract and placebo mouthwash during study period; however, the type of rinse received for the first surgery was selected by flip of a coin. Randomization of surgical side in each patient was kept unknown to surgeon and patients till the end of the study period.

### Data collection

Patients were instructed to rinse with 15 ml of mouthwash two times per day from one day after surgery till seven consecutive days. To record the pain, patients were instructed to quantify the level of their pain by using a 100 mm ruler as the visual analogue scale (VAS); 0 was no pain and 100 was severe and unbearable pain. Each VAS was recorded at the morning of 7 consecutive postoperative days before taking any analgesic medication and prior to rinsing.

Two follow-up appointments, two days and 7 days after surgery, were held after each surgery to evaluate the healing process. In addition, the patients were told to come back if they faced persistent or increasing pain.

### Statistical analysis

Appropriate descriptive statistics (including mean, frequency, range, or standard deviation) computed for each variable. To analyze data, Chi-square, independent sample t-test, and repeated measures of ANOVA were performed using SPSS software version 11.5 with the confidence interval of 95%.

## Results

Total of 44 patients met the inclusion criteria; however, one patient excluded as he did not participate second surgery during study period. The mean age of participants was 23.67 ± 4.78. There were no significant differences in age, gender, surgical difficulty, and operation time in both groups (Table 2).

**Table 2:** Demographic variables and surgical variables in two groups

**Study variable**	**Green tea**	**Placebo**	**P - value**
Sample size	43	43	-
Gender (M/F)	17/26	17/26	1.000
Surgical Difficulty Score			
5	21	19	0.896
6	17	18	
7	5	6	
Operation Time (min)	14.53 ± 5.12	15.07 ± 4.78	0.854
Age	23.67 ± 4.78	23.67 ± 4.78	1.000

**Figure 1:**
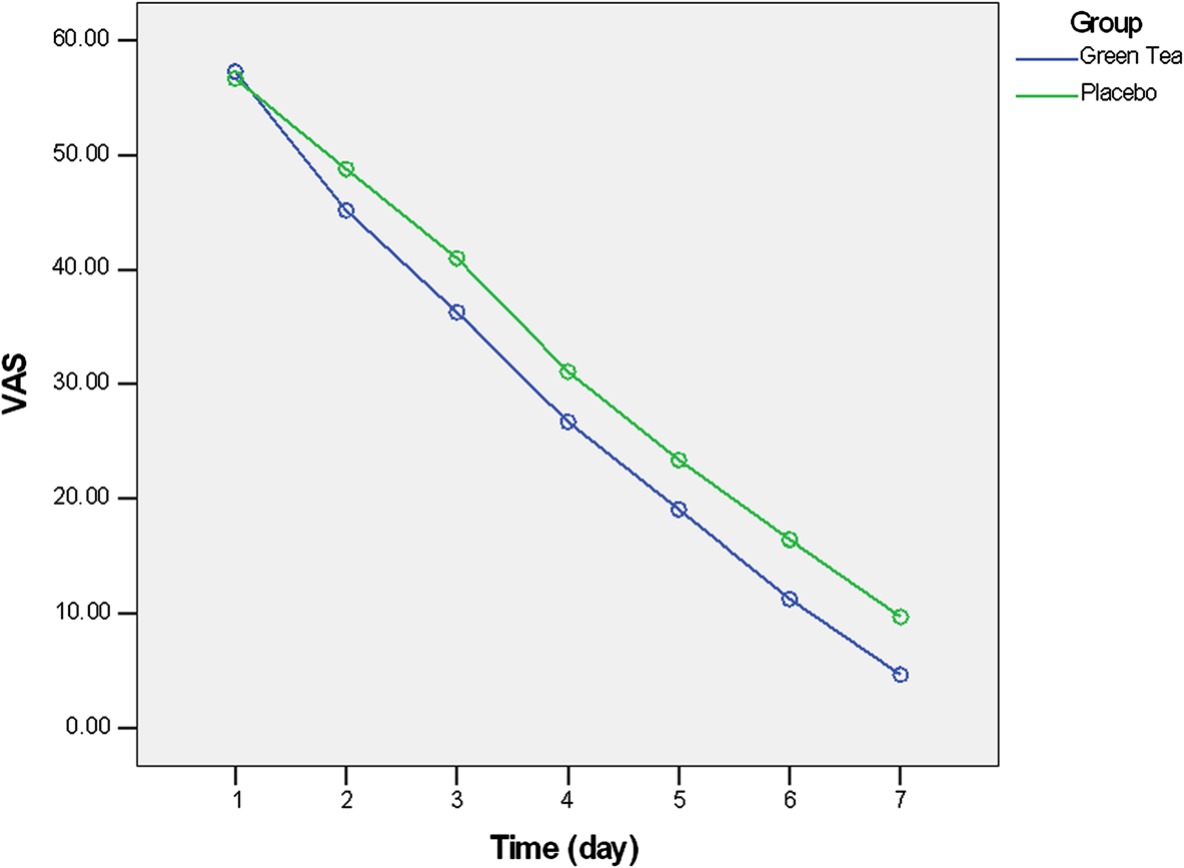
**Changes in VAS during postoperative week.** Pain was significantly lower in study group in comparison to control group during days 3–7 (P-value < 0.05).

According to the repeated measure analysis, significant difference observed in VAS values during first week after surgery (effect size = 0.971, P-value < 0.001). There were also significant changes in VAS values during the first week in both groups (effect size = 0.339, P-value < 0.001). Between groups analysis revealed that in study group the mean value of VAS was significantly lower in days 3 to 7 in comparison to control group (Table 3, Figure 1).

**Table 3:** The mean score of VAS during 7 postoperative days in study and control groups

**Day**	**Green tea**		**Placebo**		**P-value**
	**Mean**	**SD**	**Mean**	**SD**	
1	57.26	10.13	56.65	10.27	0.784
2	45.16	8.91	48.79	9.48	0.071
3	35.26	8.01	40.98	9.00	0.012
4	24.72	7.01	31.11	8.11	0.009
5	18.05	6.42	23.37	7.90	0.007
6	10.25	5.15	16.42	7.34	< 0.001
7	4.03	3.99	9.69	6.66	< 0.001

**Figure 2:**
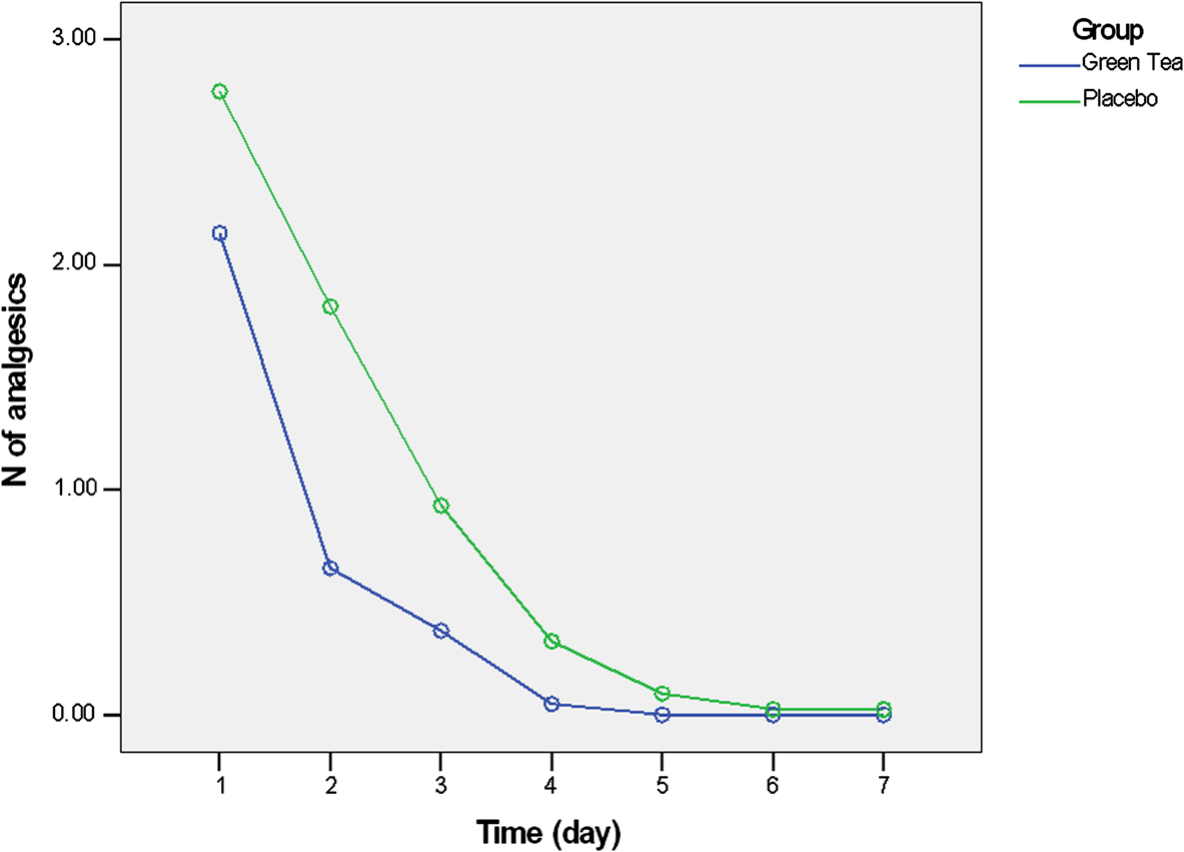
**Number and time of analgesics used.** Patients took significantly lower number of analgesics during days 1–5 when rinsing with green tea in comparison to placebo (P-value < 0.05).

In addition, a significant decrease observed in number of analgesic tablets used in both groups over time (effect size = 0.910, P-value = 0.001). Also significant changes observed in number of analgesics during 7 postoperative days when considering grouping (effect size = 0.478, P-value = 0.003). Moreover, the number of analgesics used in green tea group was significantly lower than the control group in days 1–5 after surgery (Table 4, Figure 2). Most of the analgesics (80.2%) were used during first two days after surgery in both groups.

**Table 4:** The mean number of analgesics used during 7 postoperative days in study and control groups

**Day**	**Green tea**		**Placebo**		**P-value**
	**Mean**	**SD**	**Mean**	**SD**	
1	2.14	0.41	2.77	0.78	< 0.001
2	0.65	0.69	1.81	0.66	< 0.001
3	0.37	0.49	0.93	0.59	0.001
4	0.05	0.21	0.32	0.47	0.002
5	0.00	0.00	0.09	0.29	0.041
6	0.00	0.00	0.02	0.15	0.323
7	0.00	0.00	0.02	0.15	0.323

No side effects following rinsing with green tea or placebo mouthwashes reported.

## Discussion

The aim of this study was to evaluate the effectiveness of green tea mouthwash on postoperative pain of impacted third molar surgery. The study hypothesis was rejected as the pain experienced by patients was significantly lower in study group when compared to control group.

Pain following surgical removal of impacted molars is a common complication which starts few hours after surgery as the effect of local anesthesia has eliminated. To relief this pain, various protocols have been proposed; none of which was based on herbal medicine [1,7-13,21-29].

The mechanism of postoperative pain has dedicated to trauma during surgery as it increases biochemical mediators of pain and inflammation including prostaglandins, histamine, bradykinine, and serotonin [1]. These mediators initiate the inflammatory process. As a result, anti-inflammatory drugs, including NSAIDs, are commonly prescribed to control postoperative pain [30,31]. However, peptic ulcer, gastrointestinal bleeding or perforation, renal function impairment, and platelet function inhibition are of possible side effects mentioned for NSAIDs [14].

Aromatic components of green tea have shown anti-inflammatory properties at the site of inflammation [15,32]. Results of the current study indicated that green tea extract effectively reduced the postoperative pain after initiation of rinsing. This could be dedicated to anti-inflammatory action of green tea components at the site of surgery.

In addition to inflammation, bacterial infection following impacted molar surgery increases postoperative pain [22,25]. Catechins of green tea (EGC, EGCg, and ECg) possess antibacterial activities and in vitro and in vivo studies have reported effectiveness of green tea against bacteria in periodontal diseases and caries [1,16]. Hence, the results of the current study could be also attributed to antibacterial properties of green tea mouthwash along with its anti-inflammatory activity.

The effectiveness of green tea mouthwash was in consistent with studies using antibiotics, chlorhexidine mouthwash, or low level laser therapy (LLLT) which have shown significant reduction in postoperative complications including pain [22,24-29]. However, rinsing with green tea does not possess the side effects of antibiotics (including bacterial resistancy) and chlorhexidine (including taste changes and oral discoloration). Moreover, green tea is commonly available in eastern countries and its accessibility and cost make it more appropriate in such countries in comparison to low level laser therapy.

This study was double blind as either the surgeon or the patient had no idea about rinsing solution. In addition, as the study was split-mouth, each patient served as his/her own control. This design led to elimination of age and gender as confounding variables. We also controlled for other confounding factors including difficulty of surgery, operation time, surgeon experience, and number of analgesics used after surgery.

Previous reports state that amount of trauma during surgery affect the magnitude of postoperative pain directly [25,33]. Two indices of trauma in molar extraction surgery are extraction difficulty score (based on radiograph) and operation time [33]. In the current study both factors had no significant differences in study and control group. Moreover, experience of surgeon could also affect the amount of trauma and hence postoperative complications [34]. This factor also eliminated as all the surgeries performed by one surgeon.

According to the results of this study, number of patients used analgesics and also number of analgesics taken, were lesser in study group in comparison to control group. This could be dedicated to effectiveness of green tea in controlling postoperative pain. In addition, as the greater proportion of analgesics was taken during first two days after surgery and control group used higher number of analgesics, the insignificant difference in VAS of second day in two study groups may be related to analgesic drugs.

Pain has a subjective nature and there exists difficulties to measure it. Seymour et al. reported that visual analogue scale (VAS) is a sensitive and reliable tool to evaluate the pain following surgical extraction of impacted molars [35]. This scale has been used widely in the studies that investigate the effectiveness of pain interventions after oral surgeries [36]. According to the essence of pain, in the most of the studies the personal differences in participants play as a confounding factor [36]. However, in the current study the pain scale of each participant compared to his/her own pain following rinsing with two types of mouthwash.

There were also some limitations in the current study. Rinsing with mouthwash during the day of surgery lead to blood clot resolution; hence, we had to prescribe analgesics at the day of surgery – as the postoperative pain reaches highest level 6–12 h after surgery – and instruct patients to start rinsing at the first postoperative day. This limitation is under research as investigators have designed a study to use green tea extract via slow releasing systems that is usable immediately after surgery.

## Conclusion

The results of the current study indicated that daily rinsing with green tea may be beneficial to control postoperative complications of impacted molar surgery including pain. Moreover, the need for analgesics would become less and side effects following using antibiotics, NSAIDs, or chlorhexidine mouthwash could be escaped.

## Competing interests

The authors declare that they have no competing interests.

## Authors’ contributions

ME and AN designed the study and wrote the study protocol. ME performed surgeries. NMR provided VAS and collected demographic and surgical data. AN performed randomization and kept it secret. HM analyzed the data and performed literature review. All authors participated in drafting, critical evaluation, and approval of final version of manuscript. All authors read and approved the final manuscript.
